# Tumor dosimetry using ^177^Lu: influence of background activity, measurement method and reconstruction algorithm

**DOI:** 10.1186/s40658-023-00561-8

**Published:** 2023-06-21

**Authors:** Peter Frøhlich Staanum

**Affiliations:** Department of Nuclear Medicine and PET-Centre, Aarhus University Hospital, Palle Juul-Jensens Boulevard 165, 8200 Aarhus N, Denmark

**Keywords:** Radionuclide therapy, Tumor dosimetry, Lu-177, Phantom

## Abstract

**Background:**

Image-based tumor dosimetry after radionuclide therapy, using the isotope ^177^Lu, finds application e.g., for tumor-to-organ dose comparison and for dose response evaluation. When the tumor extent is not much larger than the image resolution, and when ^177^Lu is found in nearby organs or other tumors, an accurate determination of tumor dose is particularly challenging. Here a quantitative evaluation of three different methods for determining the ^177^Lu activity concentration in a phantom is performed, and the dependence on a variety of parameters is described. The phantom (NEMA IEC body phantom) has spheres of different size in a background volume, and sphere-to-background ^177^Lu activity concentration ratios of infinity, 9.5, 5.0 and 2.7 are applied. The methods are simple to implement and well-known from the literature. They are based on (1) a large VOI encompassing the whole sphere, without background activity and with volume information from other sources, (2) a small VOI located in the sphere center, and (3) a VOI consisting of voxels with voxel value above a certain percentage of the maximum voxel value.

**Results:**

The determined activity concentration varies significantly with sphere size, sphere-to-background ratio, SPECT reconstruction method and method for determining the concentration. Based on the phantom study, criteria are identified under which the activity concentration can be determined with a maximal error of 40% even in the presence of background activity.

**Conclusions:**

Tumor dosimetry is feasible in the presence of background activity using the above-mentioned methods, provided appropriate SPECT reconstructions are applied and tumors are selected for dosimetry analysis according to the following criteria for the three methods: (1) solitary tumor with diameter > 15 mm, (2) tumor diameter > 30 mm and tumor-to-background ratio > 2, and (3) tumor diameter > 30 mm and tumor-to-background ratio > 3.

## Background

In radionuclide therapy using the isotope ^177^Lu, e.g., peptide-receptor radionuclide therapy (PRRT) of neuroendocrine tumors [1, 2] using [^177^Lu]Lu-DOTATOC or [^177^Lu]Lu-DOTATATE, or PSMA radioligand therapy (RLT) of metastatic prostate cancers [3] using [^177^Lu]Lu-PSMA, post-therapy dosimetry of organs and tumors is feasible based on quantitative gamma-camera or SPECT imaging of the gamma emissions from ^177^Lu. Image-based organ dosimetry, in particular of kidneys, have been reported now for several years, and to some extent also tumor dosimetry [4–6]. Tumor dosimetry finds application e.g., for comparison of tumor and normal organ doses and for dose response evaluations.

In tumor dosimetry, a number of pertinent challenges must be considered, e.g., that tumors are often not visible on the non-contrast CT-scan of a SPECT/CT protocol, that partial volume effects (spill-out) can be very significant for smaller tumors, and that ^177^Lu in nearby organs or other tumors may significantly influence the measurement of ^177^Lu activity concentration in tumors (spill-in). For example, in PRRT, quantification of ^177^Lu in liver metastases will be affected by physiological uptake in the liver [7], and quite generally quantification of lesions in the abdominal region will be affected by uptake in the liver, kidneys and spleen. Restriction of a tumor dosimetry analysis to well-isolated tumors severely limits the number of lesions available for analysis, and it may in practice reduce the analysis to certain classes such as bone or lymph node metastases, which can be found far from the abdominal region.

The mean tumor dose can be calculated from measurements of the ^177^Lu activity concentration in tumors at various time points after injection. A variety of methods have been employed to determine the activity concentration based on planar scintigraphy, SPECT/CT scans, or a hybrid thereof, which address or circumvent the above-mentioned inherent challenges. A volume-of-interest (VOI) defined by a threshold value has been applied together with partial volume correction [8–10] for tumors separated from adjacent lesions. Manual placement of a small spherical VOI within a tumor [11], or of a large VOI containing the tumor with volume information from a PET/CT scan [12, 13] circumvent the need for partial volume correction. These three methods are relatively straightforward to implement, not very time-consuming and do not require an accurate delineation of the tumor. Other methods include manual delineation [14, 15] or an advanced software-based segmentation based on both planar, SPECT and CT images [16]. In a very recent proposal, lesion-volume and background dependent calibration factors were determined with the intent to apply these for correction of spill-in and spill-out [17], but this method has currently not been applied to patient data.

The aim of this paper is to obtain practically applicable limits on tumor size and tumor-to-background ratio, to aid in the selection of tumors for dosimetry analysis based on post-therapy SPECT/CT images. A phantom study was performed to evaluate to which extent the mean ^177^Lu concentration measured in different sized tumors is influenced by a variable background activity for a quantitative SPECT protocol. This was considered for the three methods employing (1) a large VOI around a tumor with knowledge of the volume [12, 13], (2) a small spherical VOI within the tumor [11] or (3) a threshold-generated VOI with partial volume correction [8]. On the basis of these phantom data, limits on tumor size and tumor-to-background activity ratio are suggested, for which the accuracy is judged satisfactory to provide useful results in tumor dosimetry.

## Methods

### Phantom

A National Electrical Manufacturers Association (NEMA) NU-2 2001 image quality phantom [18] with six fillable spheres (inner diameters 10, 13, 17, 22, 28 and 37 mm; volumes 0.52, 1.15, 2.57, 5.58, 11.49 and 26.52 ml) in a 10.2 L background volume was used for the study. The spheres were filled with a solution prepared from 2.0 GBq of non-carrier added [^177^Lu]LuCl_3_ in an excess amount of Ca-DTPA (400 mg in 2 ml, i.e., > 10^6^ DTPA molecules per LuCl_3_ molecule) diluted in isotonic water to a ^177^Lu concentration of 514 kBq/ml. The Ca-DTPA acted as a chelator to prevent ^177^Lu from sticking to the phantom walls [19]. The background volume was first filled with non-radioactive water. This phantom was scanned as described below, and then ^177^Lu in solution was added stepwise to the background volume, and the phantom was scanned with sphere-to-background ratios of 9.5:1, 5.0:1 and 2.7:1. Each time ^177^Lu was added to the background volume, the NEMA phantom was turned and shaken and then allowed to homogenize by Brownian motion in the solution for at least 8 h. In the following, these phantoms are referred to by their sphere-to-background ratio as P_∞_ (non-radioactive water background), P_9.5_, P_5.0_ and P_2.7_, respectively.

### Quantitative SPECT/CT scans

Quantitative SPECT (QSPECT) scans with voxel values representing the ^177^Lu activity concentration were obtained using a Siemens Symbia T-16 SPECT/CT scanner (Siemens Medical Solutions Inc., USA) and reconstruction using ordered-subset-expectation–maximization (OSEM) in Siemens Flash3D. The acquisition and reconstruction protocols followed our procedure for kidney dosimetry, as described in detail in Ref. [20]. Briefly, acquisition was performed with medium-energy collimators, an energy window at 208 keV (20% width), 128 × 128 matrix (4.8 mm pixel size), 64 views in total and time/view varied as described below. Lower energy windows enabled scatter and deadtime correction. Reconstruction was performed with 4 iterations, 8 subsets and corrections for attenuation, scatter and collimator blurring. For this QSPECT procedure, no post-filter was applied. Calibration, i.e., determination of sensitivity and deadtime constant, was performed 31 months prior to this phantom study, using ^177^Lu (84–3188 MBq) in closed containers placed in a scatter medium (eight bags each containing 500 ml of saline). The sensitivity was 1.09 × 10^–5^ s^−1^ Bq^−1^ and the deadtime 0.5 µs for the applied SPECT system. Stability of sensitivity was verified using ^177^Lu sources with known activity. Conversion coefficients (conversion from reconstructed voxel values to ^177^Lu concentration) depended on the time/view and were adjusted accordingly for each scan.

Scans of P_∞_ were performed three times to evaluate reproducibility, while P_9.5_ and P_5.0_ was scanned once and P_2.7_ was scanned twice. The SPECT/CT scans were performed over a 3-day period, and the time per view was adjusted to obtain a similar number of counts from the spheres in each scan, starting with 90 s/view for P_∞_ 3 h after the measurement of ^177^Lu activity.

**Fig. 1:**
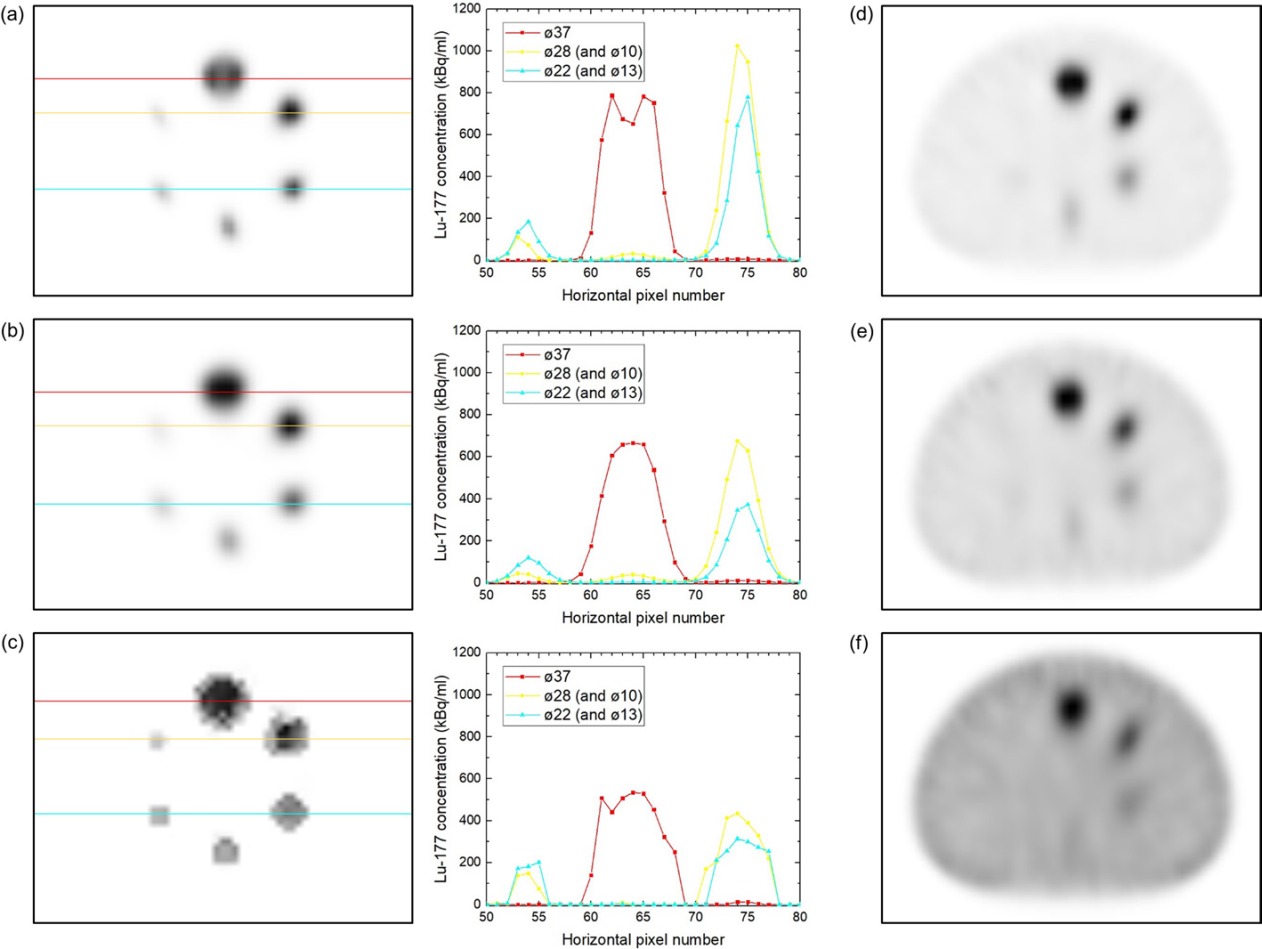
**a–c** Transverse slices and line profiles of three different reconstructions for P_∞_, the standard QSPECT OSEM reconstruction in Siemens Flash 3d (no post-filter) (**a**), the OSEM reconstruction in Siemens Flash 3d with 10 mm Gaussian post-filter (fQSPECT) (**b**) and the Bayesian reconstruction with Bowsher prior penalty factor in Hermes Hybrid Recon (**c**). **d–f** Transverse slices of P_9.5_, P_5.0_ and P_2.7_, respectively, for the QSPECT reconstruction

In the largest sphere of P_∞_, an image artifact appeared as a 'tunnel' of low intensity through the center, which was seen in the transverse plane as a central 'valley', see Fig. 1a, presumably due to resolution recovery (point-spread function correction) applied in the reconstruction [21]. In order to perform analysis without such an artifact, we performed also smoothed quantitative reconstructions by applying a 10 mm Gaussian post-filter to the (unfiltered) QSPECT reconstruction, see Fig. 1b. This reconstruction is referred to as fQSPECT ('filtered QSPECT') in the following. Also a Bayesian reconstruction with Bowsher prior penalty factor was performed in Hermes Hybrid Recon (version 1.0.30, 'AMAP' reconstruction method, 4 most similar neighbors and Bayesian weight 0.3, 4 iterations, 16 subsets with attenuation and scatter correction and resolution recovery [21]; Hermes Medical Solutions AB, Sweden), see Fig. 1c, as this was previously shown to remove the observed artifact [21]. The Bayesian reconstruction with Bowsher prior seeks to obtain SPECT images with similar voxel values in anatomically similar voxels, i.e., voxels with Hounsfield Units (HU) close to each other in the CT scan. No further scaling was applied to the fQSPECT reconstruction, as the only difference from the standard QSPECT reconstruction was the 10 mm Gaussian post-filter, and it could be verified that the total counts in the reconstructed images were essentially identical (deviation < 1.1% for P_∞_ and ≤ 0.03% for all other phantoms). For the Bayesian reconstruction a quantitative measure of activity was simply obtained by scaling the voxel values with the ratio between the sum of voxel values in the unfiltered QSPECT reconstruction to the corresponding sum in the Bayesian reconstruction.

### Analysis of quantitative SPECT scans

For each scan, the three SPECT reconstructions were analyzed in PMOD 4.006 (PMOD Technologies LLC, Switzerland) to obtain several different parameters. For P_∞_ and P_2.7_ the mean and the standard deviation of these parameters were calculated. The starting point for the analysis was six spherical VOIs (VOI_dia_) with diameters equal to the sphere diameters and aligned manually with the respective spheres on the CT scan.

#### Maximum and peak values and accuracy

In each sphere the maximum voxel value and the peak value, i.e., the maximum average in a 1 cm^3^ sphere, were used as references for selecting a threshold value (see below). They were recorded within each spherical VOI (VOI_dia_), except for the peak value in the 10 mm diameter spheres, where the VOI volume was below 1 cm^3^. These values were compared to the actual mean in the spheres.

The accuracy of the reconstructions was evaluated for P_∞_ by comparing the known activity in each sphere to the reconstructed activity within a spherical VOI with diameter equal to the sphere diameter plus 15 mm (VOI_dia+15_, concentric with VOI_dia_). The 15 mm extension was chosen in order to include essentially all counts from the respective spheres without inclusion of counts from neighboring spheres. The deviation was calculated with the known activity as reference.

#### Contrast and recovery coefficient

The contrast for each sphere was calculated as

1 $$\mathrm{Contrast}=\frac{{C}_{\mathrm{dia}}-{C}_{\mathrm{bkg}}}{{C}_{\mathrm{dia}}{+C}_{\mathrm{bkg}}}$$

where *C*_dia_ is the mean concentration in the VOI_dia_, and *C*_bkg_ is the mean concentration in a large (about 4 L) VOI in the part of the phantom without spheres.

The recovery coefficients, RC, for the various sphere sizes were determined as the ratio of the activity in the VOI_dia_ to the total activity within VOI_dia+15_ [22]. For P_9.5_, P_5.0_ and P_2.7_, the total activity within VOI_dia+15_ would contain activity in the background volume, and hence the total activity was calculated as the mean of decay corrected activity within the corresponding VOIs (VOI_dia+15_) of the three SPECT/CT scans of P_∞_.

For P_∞_, the recovery coefficients for all three reconstructions were fitted by the function

2 $$\mathrm{RC}\left(d\right)=\frac{1}{1+{\left(\frac{\alpha }{d}\right)}^{\beta }}$$

where *d* is the sphere diameter [8]. Since the recovery coefficients were determined from two VOIs within the same images, the recovery coefficients should converge to unity for large spheres, as is the case for RC(*d*) in Eq. (2).

#### Mean concentration for small spherical VOIs and threshold-based VOIs

Spherical VOIs of diameter 10, 15 and 20 mm were created concentric with the VOI_dia_ for the spheres with diameter larger than or equal to 10, 15 and 20 mm, respectively. The mean concentration within the small VOIs was recorded and compared to the actual mean in the spheres.

**Fig. 2:**
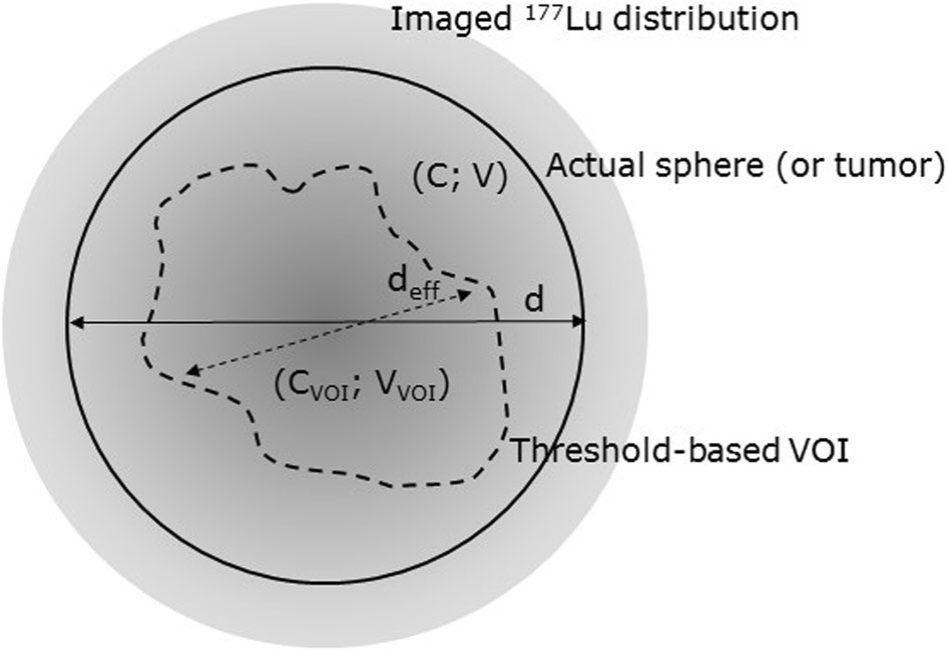
Sketch of imaged ^177^Lu distribution from ^177^Lu within a sphere or tumor (solid line) with actual, but unknown, mean concentration *C*, volume *V* and diameter *d*. The threshold-based VOI (dashed line) has mean concentration *C*_VOI_, volume *V*_VOI_ and corresponding effective diameter *d*_eff_, i.e., the diameter of a sphere with volume *V*_VOI_

Threshold-based VOIs were grown with a threshold equal to 50% and 40% of the maximum voxel value within each sphere, and also with thresholds of 60% and 50% of the peak value. The mean concentration and the volume of the VOIs were recorded. If the VOIs reached a 75 mm diameter bounding sphere, they were not evaluated further. The VOIs were generally smaller than the spheres, as shown below in the Results section, unlike in the original paper by Ilan et al. [8]. This would have made partial volume correction based directly on the VOI volume incorrect in the present work. The VOIs were instead first seen as representative volumes within the sphere, and the mean concentration in the VOIs were compared to the actual mean in the spheres. Further, to apply partial volume correction along the lines of Ilan et al. [8], the following approach was used to obtain a partial volume corrected concentration, calculated on the basis of the concentration in the VOI (*C*_VOI_) and its volume (*V*_VOI_), see Fig. 2. The true concentration in the sphere is equal to the reconstructed activity within the sphere, *A*, divided by its volume *V* and the recovery coefficient RC(*d*), see Eq. (3). The reconstructed activity *A* was approximated by the activity in the VOI (*C*_VOI_·*V*_VOI_) scaled by the volume ratio *V*/*V*_VOI_. The sphere diameter *d* was approximated as the effective VOI diameter (*d*_eff_ in Fig. 2, the diameter of a sphere with volume identical to the VOI volume) scaled by the factor *v*^1/3^, where *v* is the ratio between the sphere volume and the VOI volume. In total, the concentration

3 $$C=\frac{A}{V\cdot \mathrm{RC}(d)}\approx \frac{\left({C}_{\mathrm{VOI}}\cdot {V}_{\mathrm{VOI}}\right)\cdot V/{V}_{\mathrm{VOI}}}{V\cdot \mathrm{RC}\left({d}^{\prime}\right)}=\frac{{C}_{\mathrm{VOI}}}{\mathrm{RC}({d}^{\prime})}$$

where

4 $$d^{\prime}={\left(v\cdot \frac{6\cdot {V}_{\mathrm{VOI}}}{\pi }\right)}^{1/3}$$

*v* was determined by first performing a fit in Origin Pro 2015 (OriginLab Corporation, USA) of the function

5 $${V}_{\mathrm{VOI}}(V)={V}_{0}+c\cdot V\cdot \left(1-{e}^{-V/{V}_{1}}\right)$$

to data for the five largest spheres in P_∞_ for each threshold method. This function was chosen for its asymptotic behavior, approaching *V*_0_ for small *V* and *c*·*V* for large *V*. The constraint 0 < *c* ≤ 1 was imposed. Then *V*(*V*_VOI_) was determined from a lookup-table generated on basis of the fitted parameters, and

6 $$v=\frac{V\left({V}_{\mathrm{VOI}}\right)}{{V}_{\mathrm{VOI}}}$$

was found. When *V*_VOI_ < *V*_0_ the corresponding data point was excluded from this analysis.

#### Volume- and background dependent calibration factors

For completeness, we also carried out analysis following the procedure proposed by Raskin et al. [17], in order to investigate if this procedure would be feasible for the QSPECT protocol within the range of sphere-to-background ratios applied here. Only the unfiltered QSPECT reconstruction was considered as this was most analogous to the reconstruction used by Raskin et al. The calibration factor CF, i.e., the count rate for a VOI divided by the known activity in that VOI, was determined for each sphere as

7 $${\text{CF}}=\frac{{\text{C}}_{\text{dia}}}{{\text{C}}_{\text{true}}}\cdot S$$

where *C*_true_ is the known activity concentration in the sphere and *S* = 10.9 s^−1^ MBq^−1^ is the sensitivity determined from the QSPECT calibration procedure [20]. The so-called Sphere-to-Background Voxel Ratio (SBVR) is equivalent to *C*_dia_/*C*_bkg_ and was determined as such. CF versus SBVR and CF versus sphere volume was plotted and compared to the corresponding figures in Ref. [17].

## Results

### Maximum and peak values and accuracy

**Fig. 3:**
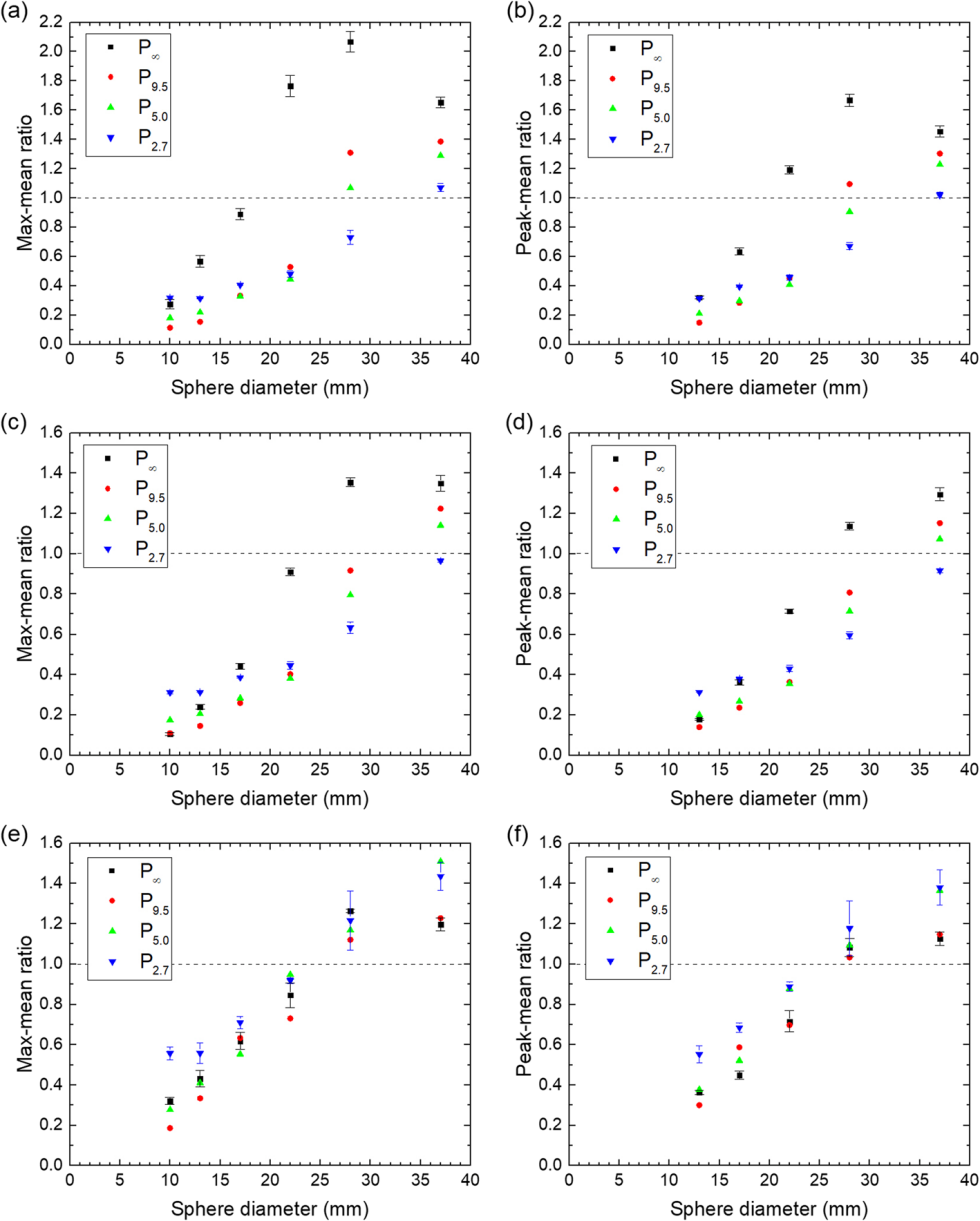
Maximum and peak values relative to mean concentration for all spheres (except peak value for ø10) in all phantoms for the QSPECT reconstruction (**a**, **b**), the fQSPECT reconstruction (**c**, **d**) and the Bayesian reconstruction (**e**, **f**). The dashed lines indicate a ratio of unity

**Fig. 4:**
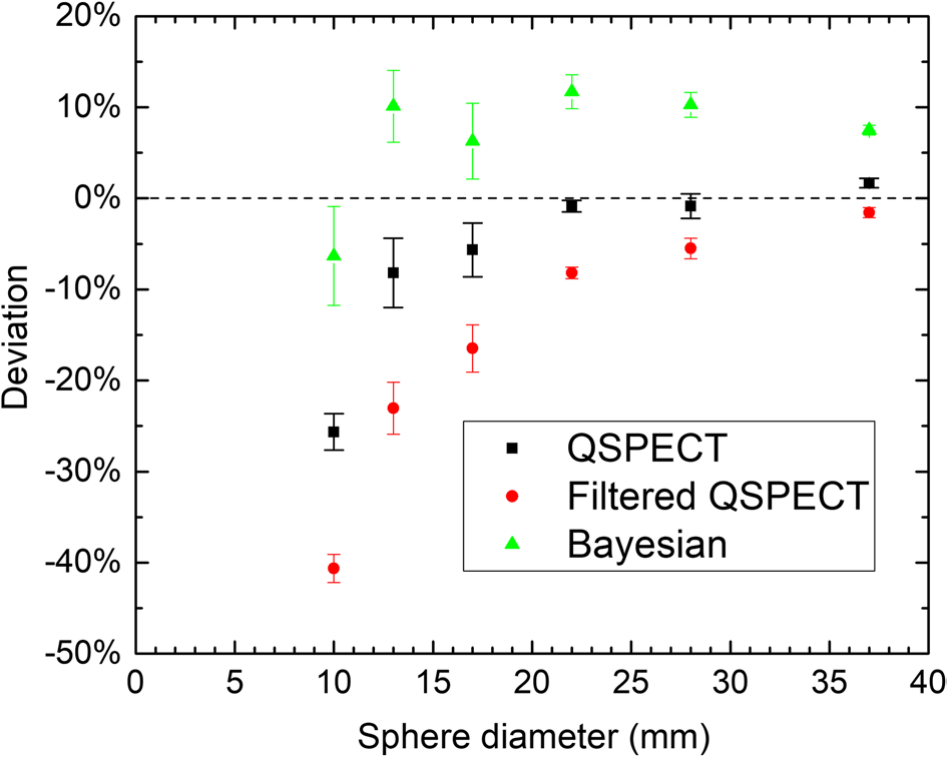
Deviation from unity ratio between reconstructed and actual activity for the three different reconstructions

The maximum and the peak values relative to the mean concentration of the spheres are shown for all spheres and phantoms in Fig. 3, while the deviation from unity ratio between reconstructed and actual activity is shown in Fig. 4.

### Contrast and recovery coefficient

**Fig. 5:**
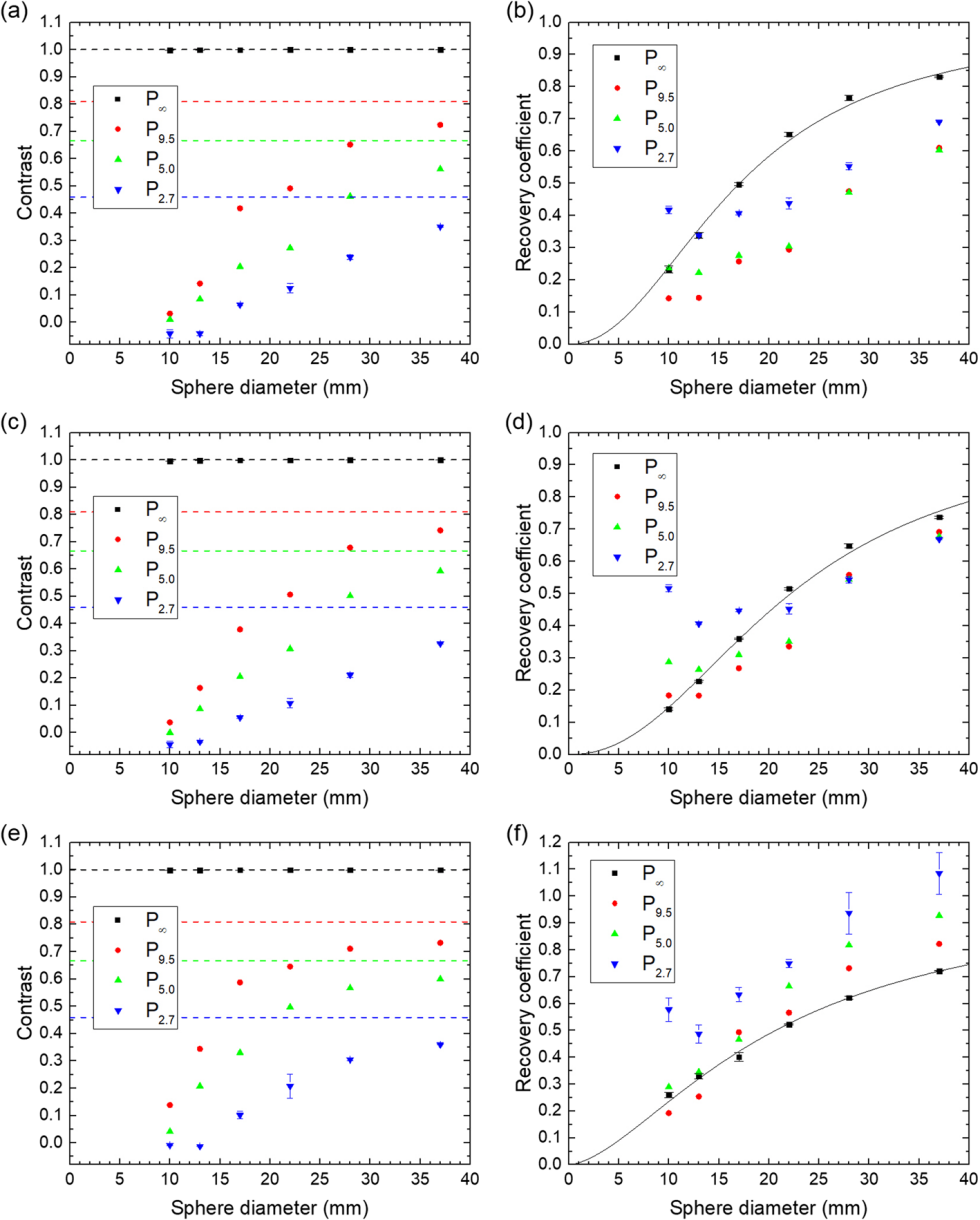
Contrast and recovery coefficients for all spheres in all phantoms for the QSPECT reconstruction (**a**, **b**), the fQSPECT reconstruction (**c**, **d**) and the Bayesian reconstruction (**e**, **f**). The dashed lines indicate the theoretical contrast values calculated from the sphere-to-background ratios and Eq. (1). The solid lines represent the fit of Eq. (2)

The contrast and the recovery coefficients are shown for all spheres and phantoms in Fig. 5. The fitted values of *α* and *β* in Eq. (2) are (*α*; *β*) = (17.1 mm; 2.15), (22.2 mm; 2.21) and (20.6 mm; 1.63) for the QSPECT, the fQSPECT and the Bayesian reconstruction, respectively.

### Mean concentration for spherical VOIs and threshold-based VOIs

**Fig. 6:**
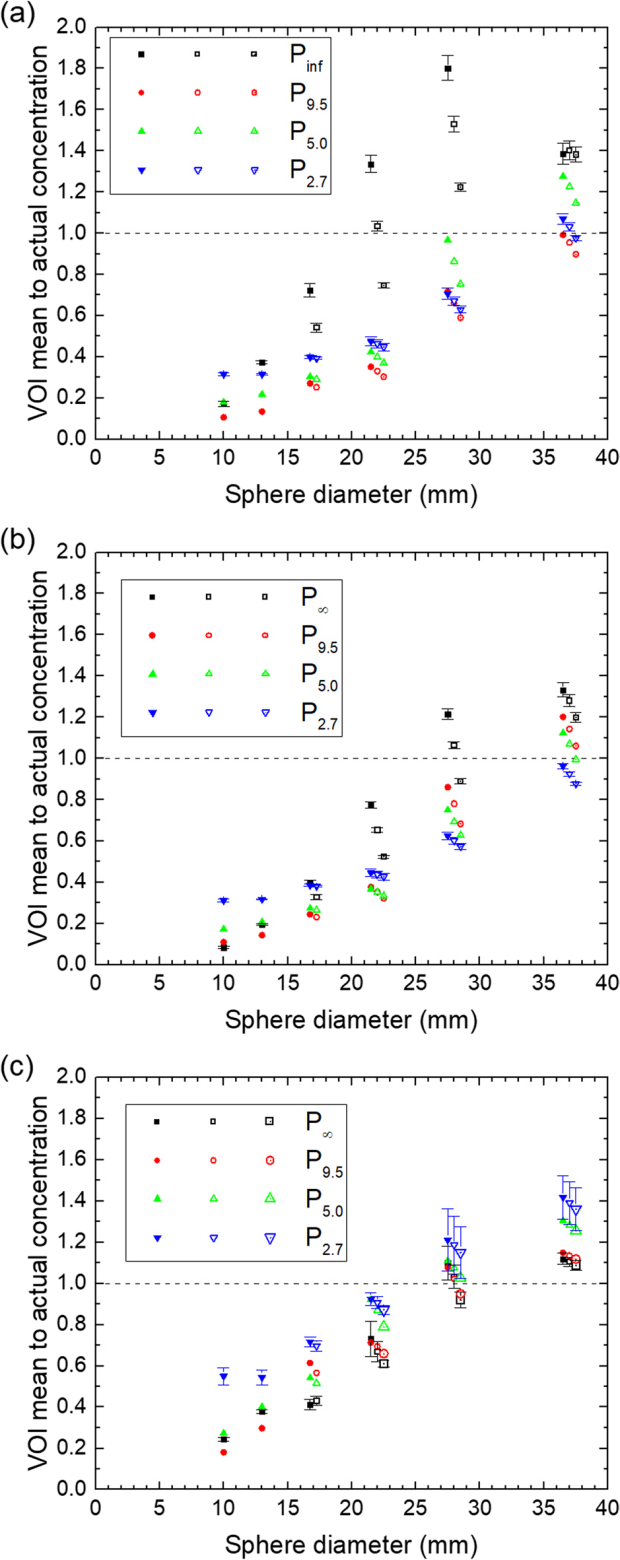
Mean to actual concentration in the spherical VOIs vs. sphere diameter for the QSPECT reconstruction (**a**), the fQSPECT reconstruction (**b**) and the Bayesian reconstruction (**c**). Solid, open and open symbols with a dot represent ø10, ø15 and ø20 VOIs, respectively. They have been displaced horizontally for clarity. The dashed lines indicate a ratio of unity

The mean concentrations in the spherical VOIs relative to the actual concentration are shown for all spheres in all phantoms in Fig. 6.

**Fig. 7:**
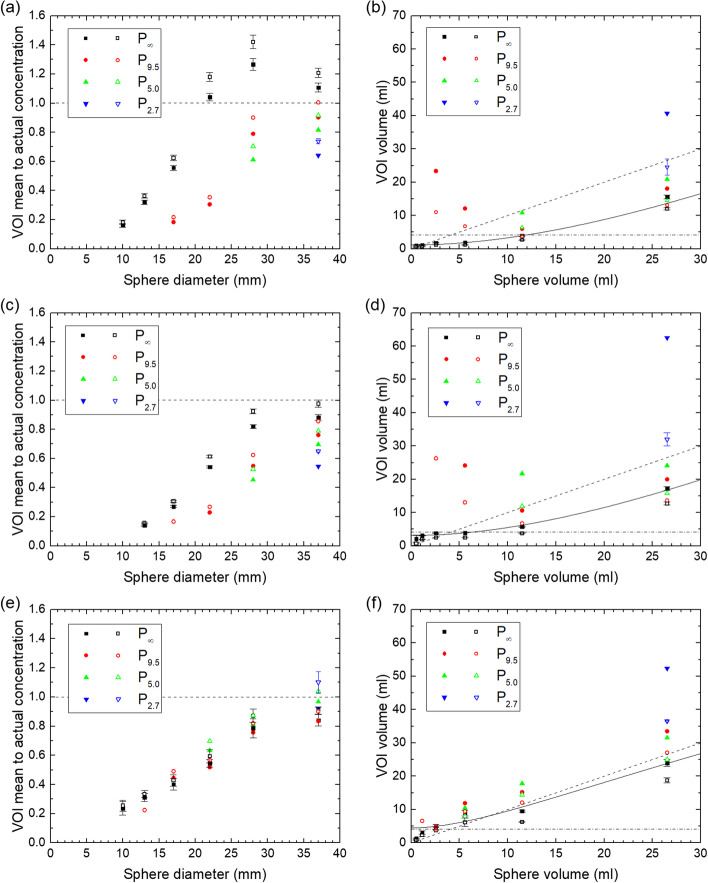
Ratio between mean concentration in the threshold-based VOIs (40% and 50% of maximum voxel value) and actual concentration and volume of these VOIs for the QSPECT reconstruction (**a**, **b**), the fQSPECT reconstruction (**c**, **d**) and the Bayesian reconstruction (**e**, **f**). Data for VOIs based on 40% and 50% of the maximum voxel value are indicated by solid and open symbols, respectively. The dashed lines in **a**, **c**, **e** indicate a ratio of unity, while in **b**, **d**, **f** they represent the lines of identity and the dash-dotted lines indicate the volume of a ø20 sphere. The solid lines indicate the fit of Eq. (5) for the 40% of maximum value method

**Fig. 8:**
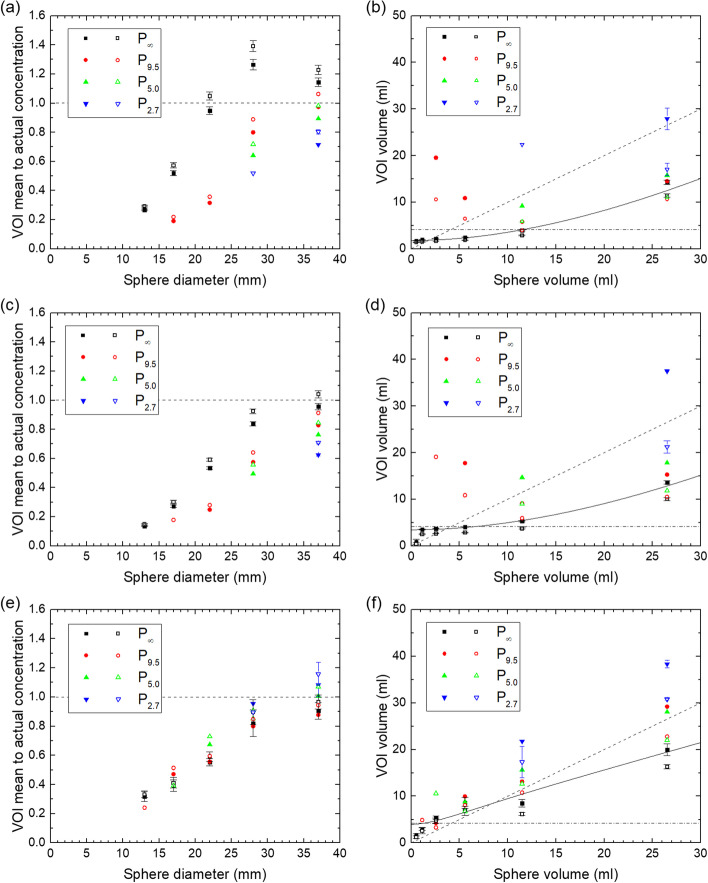
Ratio of mean concentration in the threshold-based VOIs (50% and 60% of peak value) and actual concentration and volume of these VOIs for the QSPECT reconstruction (**a**, **b**), the fQSPECT reconstruction (**c**, **d**) and the Bayesian reconstruction (**e**, **f**). Data for VOIs based on 50% and 60% of the peak value are indicated by solid and open symbols, respectively. The dashed lines in **a**, **c**, **e** indicate a ratio of unity, while in **b**, **d**, **f** they represent the lines of identity and the dash-dotted lines indicate the volume of a ø20 sphere. The solid lines indicate the fit of Eq. (5) for the 50% of peak value method

For the threshold-based VOIs, the uncorrected mean to actual concentration is shown for all spheres in all phantoms in Figs. 7a, c, e and 8a, c, e and the corresponding volumes of the VOIs are shown in Figs. 7b, d, f and 8b, d, f.

The fit of Eq. (5) resulted in values of V_0_ in units of ml of (0.656; 1.157; 1.281; 1.773) for the QSPECT reconstruction, (1.966; 3.129; 2.444; 3.457) for the fQSPECT reconstruction and (3.492; 4.499; 4.229; 3.978) for the Bayesian reconstruction given in the order (50% max, 40% max, 60% peak, 50% peak)-method. The values of V_1_ in units of ml are (48.9; 41.6; 55.9; 51.1), (60.6; 36.7; 84.9; 60.6) and (31.4; 9.5; 43.8; 3.5), respectively. The values of c are equal to the boundary of 1, except for the 40% max and 50% peak methods for the Bayesian reconstruction, where c is 0.776 and 0.584, respectively. Representative fits are shown in Figs. 7b, d, f and 8b, d, f.

**Fig. 9:**
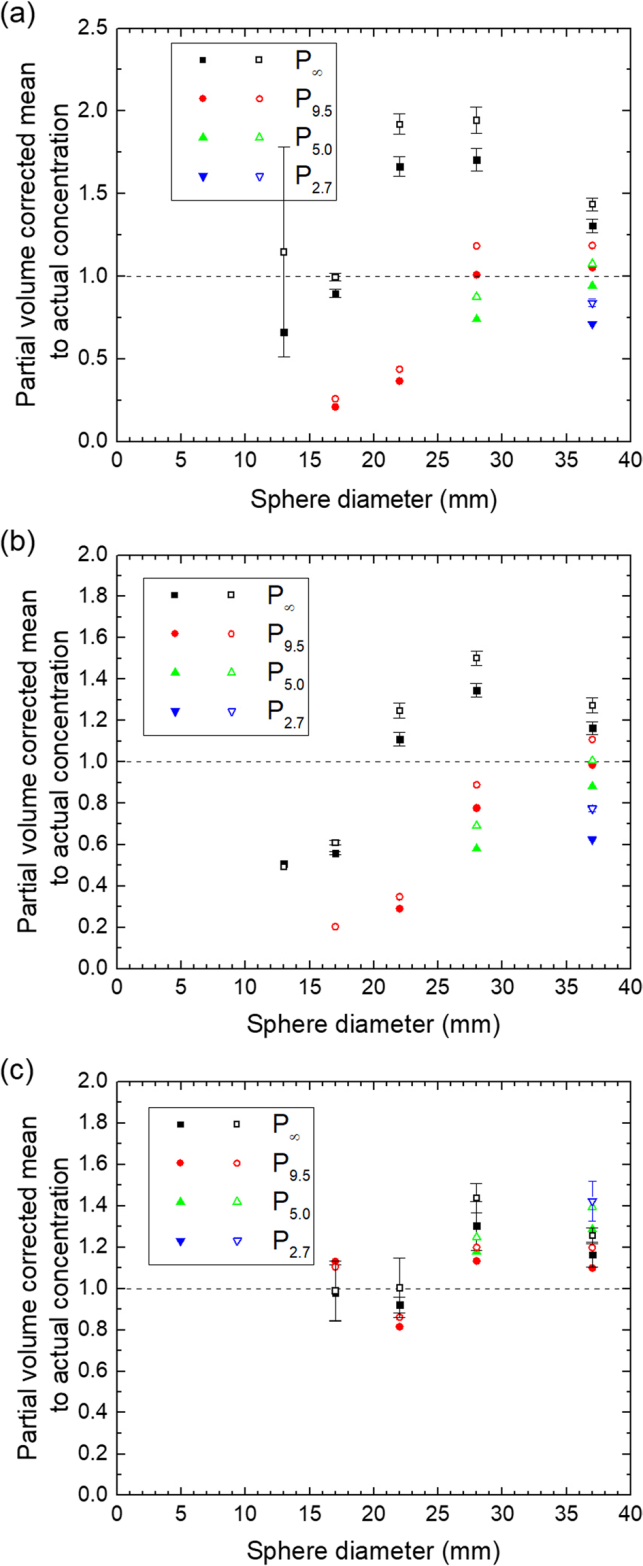
Ratio of partial-volume corrected mean and actual concentration for the QSPECT reconstruction (**a**), the fQSPECT reconstruction (**b**) and the Bayesian reconstruction (**c**). Data for VOIs based on 40% and 50% of the maximum voxel value are indicated by solid and open symbols, respectively. The dashed lines indicate a ratio of unity

**Fig. 10:**
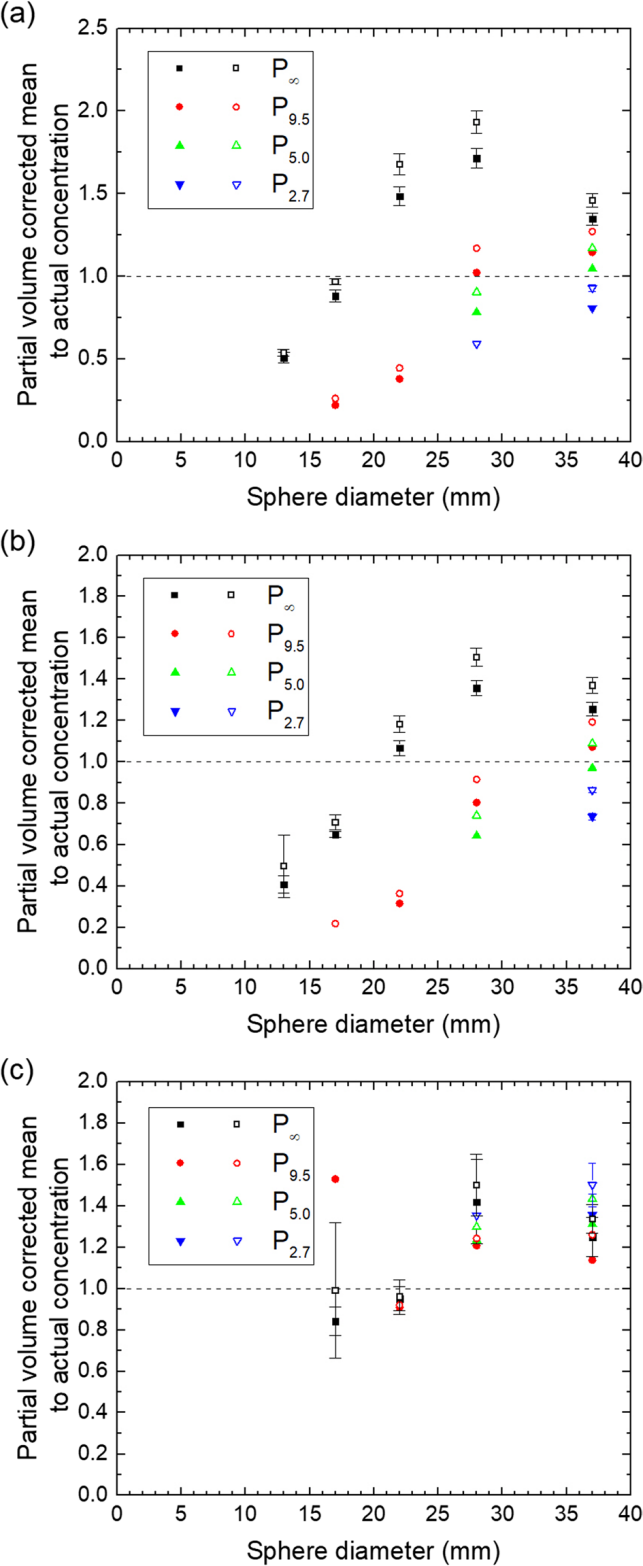
Ratio of partial-volume corrected mean and actual concentration for the QSPECT reconstruction (**a**), the fQSPECT reconstruction (**b**) and the Bayesian reconstruction (**c**). Data for VOIs based on 50% and 60% of the peak value are indicated by solid and open symbols, respectively. The dashed lines indicate a ratio of unity

The results for the mean values with correction for the partial volume effect using Eqs. (3)–(6) are shown in Figs. 9 and 10, respectively.

### Volume- and background dependent calibration factors

**Fig. 11:**
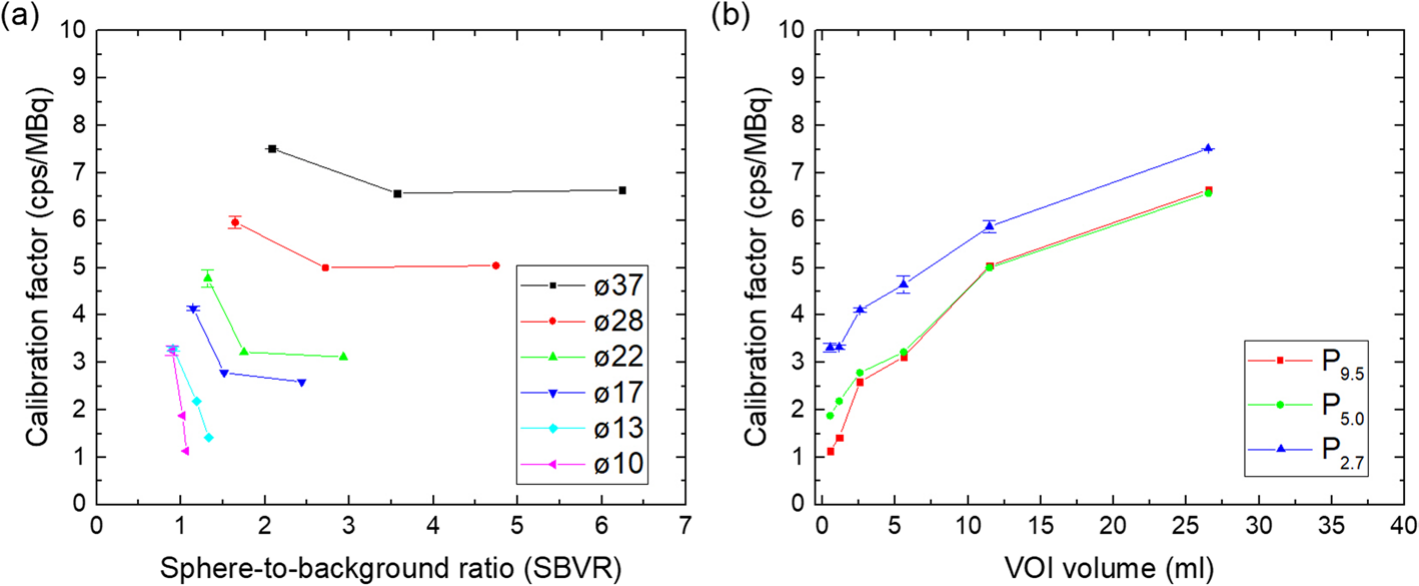
CF versus SBVR (**a**) and CF versus VOI volume (**b**) for all spheres for the QSPECT reconstruction akin to Figs. 4 and 5 in Ref. [17]. The connecting lines are only intended to guide the eye

The calibration factors are shown as a function of SBVR and VOI volume in Fig. 11 for the QSPECT reconstruction.

## Discussion

### Phantom study

At the outset this study was planned with only the routinely applied QSPECT reconstruction without post-filtering [20, 23] in order to eventually apply the same reconstruction for tumor dosimetry as for kidney dosimetry. The 'valley' artifact observed in Fig. 1a, however, reduces the maximum and the peak values in the ø37 sphere to values below those of the ø28 sphere as shown in Fig. 3a. This carries on to lower the fixed-percentage thresholds and in turn influences values derived from the threshold-generated VOIs. The artifact can probably be ascribed to Gibbs-like ringing artifacts in performing point-spread function correction as discussed by Kangasmaa et al*.* [21]. The artifact is not visible in Fig. 1d–f, where the transition from spheres to background is less sharp due to the presence of ^177^Lu in the background volume. The smoothing by a Gaussian post-filter removes the artifact, and a more regular behavior is found in Fig. 1b and in Fig. 3c, d, where the mean and peak values for the ø37 sphere are larger than or equal to the values for the other spheres. With the Bayesian reconstruction, the voxel values are similar in voxels with similar Hounsfield Units in the CT scan. Indeed, in Fig. 1c, the voxel values across the spheres vary less than in the OSEM reconstructions and there is a sharp edge to the background.

As shown in Fig. 4, the accuracy is very good for the five largest spheres using the QSPECT reconstruction, which also means that despite the 'valley'-artifact, the total counts are maintained within the ø37 sphere. For the fQSPECT reconstruction, the negative deviation is due to counts found outside the VOI_dia+15_ spheres as a consequence of the smoothing. For the Bayesian reconstruction the deviation is within 10% over the entire range of sphere sizes. A slightly better agreement may be obtainable by performing a full calibration for the Bayesian reconstruction rather than performing a scaling based on the unfiltered QSPECT reconstruction, however, this was not explored as the scaling approach was judged satisfactory for the present work.

The contrast shown in Fig. 5 is 100% for all reconstructions and all sphere sizes for P_∞_, however, once background activity is introduced the values drop below the theoretical values in particular for the smallest spheres. Visually judged from the reconstructed images, the presence of a sphere, or tumor, cannot be recognized once the contrast is below about 0.2 (compare Figs. 1d–f and 5). The RCs in Fig. 5 show a regular behavior for P_∞_, but for the other phantoms the presence of activity in the background volume causes the RCs of the smallest spheres to become larger than the RCs of some of the larger spheres. For the Bayesian reconstruction the picture is even inverted, such that the RCs for P_2.7_ and P_5.0_ are generally above those of P_9.5_ and P_∞_.

One method for calculating tumor dose is based on the mean concentration in small spherical VOIs, for which data are shown in Fig. 6. The mean is within about 40% of the true value only for the largest sphere using the QSPECT reconstruction, but for the two largest spheres using the fQSPECT reconstruction and for the three largest using the Bayesian reconstruction. The variation of the obtained mean with ^177^Lu content in the background is largest for the QSPECT reconstruction and smallest for the Bayesian reconstruction. The variation with chosen small VOI diameter can be observed from the series of three displaced data points for the same phantom and sphere diameter. Generally the mean decreases with increasing VOI diameter, as expected from the profiles in Fig. 1, where the voxel values vary across the sphere, being largest in the center, except in the presence of the 'valley'-artifact. For the smaller spheres the mean values are generally quite low as compared to the true value, which can be explained by the low RCs and that the spherical VOIs probe a large fraction of the sphere volume.

For the method based on threshold-generated VOIs, the uncorrected data presented in Figs. 7a, c, e and 8a, c, e show that for the QSPECT reconstruction, values within 50% of the true value are obtained for the two largest spheres, while the mean is generally underestimated for the other two reconstructions except for the ø37 sphere. For the QSPECT reconstruction with a 40% of maximum or 50% of peak threshold, values within 40% of the true value are obtained for the two largest spheres for all phantoms except P_2.7_. The mean values in VOIs generated by a percentage of the peak value were expected to be less susceptible to noise than those generated from the maximum in a single voxel, however, no appreciable difference in standard deviation of the mean is seen between the two. The volumes probed by the VOIs, as shown in Figs. 7b, d, f and 8b, d, f, vary significantly with the level of ^177^Lu background activity, and in particular for the Bayesian reconstruction the volume may extend outside the sphere volume.

A model for partial volume correction was presented, and to this end the function in Eq. (5) was fitted to the volumes V_VOI_ vs V. The parameter c is not well determined, probably due to the lack of data points in the asymptotic limit of large volumes, and in most cases it is limited to the boundary of 1. Opportunely, the most important point is a good approximation at intermediate values of sphere volume, or diameter, where the slope of RC(*d*) is largest, which is also within the range where data points were obtained. Equation (5) was fitted to data points for P_∞_, and as *V*_VOI_ was found to increase with background activity concentration, the scale factor *v* will be overestimated in the presence of background activity. This implies an overestimate of RC(*d*') and hence an underestimate of the correction 1/RC(*d*'). Additionally, the decrease found in RC(*d*) for increasing background activity for the OSEM reconstructions in Fig. 5 also leads to an underestimate of the correction, when RC(*d*) for P_∞_ is applied. One might argue, that by lowering the threshold one could obtain threshold-based VOIs with volume equal or close to the sphere volumes, in order to enable partial volume correction directly on the basis of the VOI volume. This is true for P_∞_, but in the presence of background activity or in the clinical case of an inhomogeneous background, the VOIs would in many cases grow unbounded by the sphere or tumor.

With correction for the partial volume effect, as presented in Figs. 9 and 10, the QSPECT reconstruction performs not very well with deviations between 50 and 100% even for the ø22 and ø28 spheres in P_∞_. The Bayesian reconstruction on the other hand is very stable across all sphere sizes as compared to the other reconstructions. The values for the fQSPECT reconstruction with a threshold at 40% of the maximum or 50% of the peak value are within 40% of the true value for P_∞_ with a sphere diameter of 22 mm and larger. This is in line with the procedure applied by Ilan et al. [8], using a 42% threshold and a similar QSPECT procedure, with the same number of iterations and subsets in the OSEM reconstruction as here and a Hanning filter with cutoff at 0.85 cycles/cm. Once ^177^Lu activity is introduced to the background volume, the corrected mean drops significantly for the ø22 sphere (P_9.5_) or the threshold-based VOI even extends beyond limits (P_5.0_ and P_2.7_), while for the ø28 and ø37 spheres the mean remains within about 40% of the true value.

In Fig. 11a a linear increase of CF as a function of SBVR was anticipated on the basis of Fig. 4 in the paper by Raskin et al. [17], but this is clearly not the case. Apparently this linear behavior breaks down for the lower sphere-to-background ratio applied here (actual 2.7–9.5; SBVR 1.3–6.2 for the three largest spheres) as compared to Ref. [17] (actual 6–17; SBVR 2.4–14.6)—at least for the applied acquisition and reconstruction parameters. For the two smallest spheres, not analyzed in Ref. [17], a marked decrease of CF with SBVR can even be observed. The behavior of CF with respect to volume as shown in Fig. 11b is similar to the measured data points in Fig. 5 of Ref. [17], however, the CF is found to increase with decreasing SBVR (due to spill-in), while the opposite behavior is seen in Ref. [17], possibly due to the larger number of OSEM iteration updates (100 vs. 32 in the present work). We further note that the CF is strongly dependent on volume for the considered sphere volumes, which would make a correction rather sensitive to the determined volume of a given lesion. With the significant differences found in comparison to Ref. [17], further investigations would be needed to apply the method of Raskin et al. for correction of volume- and background-dependence.

### Translation to tumor dosimetry

For tumor dosimetry, we assume that the mean tumor dose is calculated from the mean ^177^Lu activity concentration in a tumor in sequential post-treatment scans, and that the beta-radiation is completely absorbed within the tumor.

When the results of the phantom study are considered in the context of tumor dosimetry, some options and limitations become clear. The data obtained for P_∞_ in Fig. 4 indicate, that the ^177^Lu activity in a well-isolated tumor even down to 13 mm diameter can be determined to within about 10% using a large VOI around the tumor on the QSPECT reconstruction. In clinical cases, typical quantification accuracy of ^177^Lu activity is about 10–20% [20, 23, 24], and provided the tumor volume is determined with a similar accuracy using other imaging methods, e.g. PET, CT or MR, a similarly accurate measure of ^177^Lu concentration can be obtained.

The methods based on a small VOI or a threshold-generated VOI can be applied not only for isolated tumors, but also if nearby activity is present, e.g. in other tumors or as physiological uptake in liver, spleen or kidneys. For the small VOI method and the threshold method with partial volume correction, the best agreement is found for the two or three largest spheres for the fQSPECT and the Bayesian reconstruction, respectively, with the threshold set to 40% of the maximum or 50% of the peak value. We also observed that for the QSPECT reconstruction a threshold set to 40% of the maximum or 50% of the peak value leads to equally good agreement without partial volume correction. This is an empirical observation, and the method can be seen as an alternative to the small VOI method for selecting a representative volume. For practical reasons it could be an attractive option, as it makes use of the QSPECT reconstruction, which already is applied for kidney dosimetry.

By the nature of the Bayesian reconstruction, it critically depends on the alignment between SPECT and CT, and hence the relatively good performance in this phantom study cannot necessarily be translated to patient studies, e.g. an 'erratic fragmented appearance' of lesions was reported by Grootjans et al. [25], and the Bayesian reconstruction should only be used with caution. Hence, the small VOI or the threshold-generated VOI method are judged more robust and straightforward to apply using an OSEM reconstruction.

As a practical limit we suggest for both the small VOI method and the threshold-based methods a tumor diameter > 30 mm. This is slightly larger than the ø28 sphere and thus a bit conservative to allow for some deviations from the idealized case of a phantom study. Tumors down to 22 mm diameter can also be included for analysis, if they are well isolated, using either the small-VOI method with a ø10 VOI or the threshold-based methods. Similarly for the large VOI method, we slightly conservatively require a tumor diameter larger than 15 mm. Tumor diameters are often reported as the longest diameter in the plane of measurement [26], while partial volume effects are expected to be most significant in the plane of the shortest axis of non-spherical volumes. Hence if the aspect ratio differs significantly from unity, e.g. as for very elongated tumors, the short axis should also fulfill the relevant limit. It is well-known from phantom studies of kidneys that the recovery coefficient decrease with increasing aspect ratio, or surface-to-volume ratio, as well as with the complexity of the geometry [27, 28], and similar results would be expected from a similar study of non-spherical tumor geometries.

For all ø28 and ø37 spheres in all phantoms the measured contrast is > 0.2. This is, however, also at the limit where a tumor can be recognized, and as a practical limit for the small VOI method we propose a contrast > 0.3, or equivalently a tumor-to-background ratio larger than 2. Some variation of the mean concentration with small VOI diameter is found, particularly when no ^177^Lu background activity is present. A ø15 or ø20 diameter VOI may be preferable, as the concentration tends to be overestimated with a ø10 VOI, but a general optimal choice cannot be made as this depends on tumor diameter. A 20 mm diameter sphere was applied by Sandström and colleagues in kidney and spleen dosimetry [29], and adapted for tumor dosimetry by Del Prete et al. [11, 30]. The threshold-based methods (40% of maximum or 50% of peak value) could only be applied to spheres for which the measured contrast was > 0.5 or equivalently the tumor-to-background ratio was larger than 3. The tumor-to-background ratio is likely to vary in the days following treatment, and the contrast criteria must be fulfilled for all post-treatment scans used for tumor dosimetry. Normally the treatment drug is retained longer in tumors than in organs, which means of course that if the tumor-to-background criterion is fulfilled at the first post-treatment scan, it is most likely also fulfilled at later scans.

With these criteria for selecting tumors for analysis by one of the three methods, it should be possible to determine the mean ^177^Lu concentration at the level of 40% accuracy, or better with the large VOI method. Other significant error sources can be the volume determination (large VOI method), the partial volume correction (threshold-generated VOI method) or errors in fitting sequential data points to an exponential decay [20]. In clinical practice, quantification errors larger than the 40% found here cannot be excluded, and as further error sources also contribute to the total error of the tumor dose, a realistic boundary on the possible error is not below 50%. If the estimated tumor dose is within 50% of the true value, then the true value is within + 100%/− 33% of the estimated dose.

This seems perhaps unsatisfactory, but since tumor dosimetry is more difficult than kidney dosimetry, with its smaller volumes that are undiscernible on non-contrast CT, errors up to 50% are not surprising [10, 31]. For a symmetric triangular error distribution, a 50% maximal error corresponds to a standard deviation of 20% [20, 32], which can be compared to reported standard deviations in kidney dosimetry of 10–15% [20, 31].

This level of uncertainty in tumor dosimetry is lower than observed inter-patient or inter-tumor variability or the intra-patient variability through the course of a treatment series [11, 14, 33], and further the threshold-based method with partial volume correction has already been used to demonstrate tumor-dose response for pancreatic neuroendocrine tumors [8]. This holds promises that any of the methods with their associated restrictions are useful for e.g. dose response studies in PRRT and RLT using ^177^Lu.

These conclusions are based on a phantom study with spheres up to 37 mm diameter, but are also expected to hold for larger tumors, where partial volume effects are less significant [7]. The QSPECT reconstruction method is applied at least at three sites, including our own [11, 20, 34]. Overall similar conclusions are expectable for other hardware, acquisition or reconstruction parameters [35], but the limits on tumor diameter and tumor-to-background ratio as well as the choice of threshold or small VOI size may differ. A consistency check of the methods can be made for tumors, when more than one of the methods are applicable. Sandström et al. [29] demonstrated a good correspondence between the small VOI and the threshold-based method in dosimetry of the kidneys and the spleen.

## Conclusion

Determination of the mean ^177^Lu concentration in a tumor, as represented here by a sphere, is challenging and depends on factors such as tumor volume, reconstruction parameters, background activity and possibly artifacts. Using either

- the large VOI method on the QSPECT reconstruction, with tumor volume from other modalities, for solitary tumors larger than 15 mm diameter,
- the small VOI method on the fQSPECT reconstruction for tumors larger than 30 mm diameter and with tumor-to-background ratio > 2, or
- the threshold-based method (40% of maximum or 50% of peak value) without partial-volume correction on the QSPECT reconstruction, or with partial volume correction on the fQSPECT reconstruction, for tumors larger than 30 mm diameter and tumor-to-background ratio > 3,

a determination of mean ^177^Lu concentration in tumors with background activity can be made, which is expected to be useful in e.g., dose response studies and tumor to normal organ dose comparisons. None of the methods require accurate tumor delineation, and the determined concentration does not depend directly on tumor volume or tumor-to-background ratio—only the above-mentioned limits should be fulfilled. The methods are well-known and straightforward to implement using standard tools in commercially available software.

## Data Availability

The datasets used and/or analyzed during the current study are available from the author on reasonable request.

## Abbreviations

DTPA: Diethylenetriaminepentaacetic acid
IEC: International Electrotechnical Commission
NEMA: National Electrical Manufacturers Association
OSEM: Ordered-subset-expectation–maximization
PRRT: Peptide receptor radionuclide therapy
PSMA: Prostate-specific membrane antigen
QSPECT: Quantitative SPECT
RC: Recovery coefficient
RLT: Radioligand therapy
VOI: Volume-of-Interest
